# CD1d functions as a ligand for PIRA2 to drive macrophage activation in nonalcoholic fatty liver disease

**DOI:** 10.1038/s41419-026-08789-9

**Published:** 2026-04-27

**Authors:** Xiaosheng Tan, Qingwen Li, Zhibo Ma, Lingjuan Sun, Xiangli Zhao, Jianlin Chen, Jingzeng Wang, Xiufang Weng, Li Chen, Zhishui Chen, Peixiang Lan

**Affiliations:** 1 Institute of Organ Transplantation, Tongji Hospital, Tongji Medical College, Huazhong University of Science and Technology, Wuhan, China; 2 Key Laboratory of Organ Transplantation, Ministry of Education, Wuhan, China; 3 NHC Key Laboratory of Organ Transplantation, Wuhan, China; 4 Key Laboratory of Organ Transplantation, Chinese Academy of Medical Sciences, Wuhan, China; 5 Department of Immunology, School of Basic Medicine, Tongji Medical College, Huazhong University of Science and Technology, Wuhan, China

**Keywords:** Metabolic disorders, Innate immunity

## Abstract

Liver inflammation is a key driver of nonalcoholic fatty liver disease (NAFLD) and its progressive subtype, nonalcoholic steatohepatitis (NASH). Macrophages, as central players in the innate immune response, are crucial to disease pathogenesis; however, the upstream events that initiate their activation remain poorly defined. Here, we employed a cell-based chimeric receptor screening system and identified CD1d as a surface ligand for PIRA2. We subsequently demonstrated that CD1d stimulation activated macrophages both in vitro and in vivo. Co-immunoprecipitation assays further confirmed a direct interaction between CD1d and PIRA2. Using Pira2-deficient (*Pira2*^*−/−*^) mice, we observed significantly reduced hepatic inflammation and lipid accumulation compared to wild-type controls. Importantly, macrophage-specific Pira2 conditional knockout mice similarly exhibited reduced macrophage activation and inflammatory cytokine production in vivo, confirming a macrophage-intrinsic role of PIRA2. Mechanistically, CD1d-PIRA2 interaction involves the α1 and α2 domains of CD1d and the D1 and D2 domains of PIRA2, leading to FcRγ ITAM tyrosine phosphorylation and downstream inflammatory signaling-events that are impaired in *Pira2*^*−/−*^ macrophages. Additionally, CD1d and LILRA2 protein levels were elevated in NAFLD patients, and CD1d stimulation induced proinflammatory cytokine expression in human macrophages, which was attenuated by LILRA2 blockade. A recombinant LILRA2/Fc fusion protein effectively blocked CD1d-induced inflammatory gene expression in human macrophages, highlighting its potential as a therapeutic strategy for NAFLD. Collectively, our findings identify CD1d as a functional ligand of PIRA2 that promotes macrophage activation and inflammation, contributing to inflammatory progression in NAFLD.

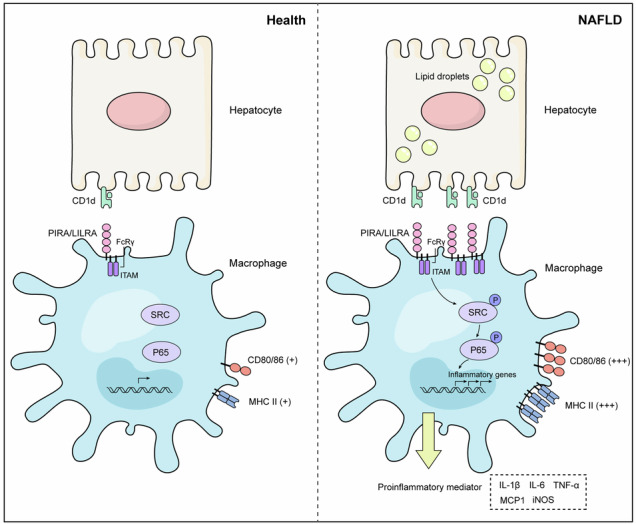

## Introduction

Nonalcoholic fatty liver disease (NAFLD), marked by the excessive accumulation of lipid droplets in the liver, has emerged as a growing global health concern. It is now one of the leading causes of liver-related morbidity, liver failure, and liver transplantation [1–3]. Despite its clinical significance, the mechanisms underlying the initiation and progression of NAFLD remain incompletely understood [4]. Multiple risk factors contribute to its pathogenesis, among which immunological elements—particularly the innate immune system—have gained increasing attention, especially in the context of its inflammatory subtype, nonalcoholic steatohepatitis (NASH) [1, 5, 6].

The liver functions not only as a metabolic hub but also as an immune organ rich in diverse immune cells. Among them, liver-resident macrophages (Kupffer cells) and infiltrating macrophages play essential roles in NAFLD pathogenesis [7, 8]. Accumulating evidence indicates that macrophages are increased and activated in children and young adults with more severe forms of NAFLD, implying that macrophage activation precedes inflammation and fibrosis and may serve as a predictor of disease progression [9, 10]. The importance of macrophages is further supported by studies showing that their depletion in mouse models of NAFLD mitigates disease development [11]. Macrophage activation can be triggered by numerous intrahepatic and extrahepatic stimuli, including fatty acids, cholesterol and its derivatives, endotoxins, translocated bacteria, and damaged hepatocytes [12–14]. which engage pattern recognition receptors (PRRs) such as Toll-like receptors (TLRs), NOD-like receptors (NLRs), RIG-I-like receptors, and C-type lectin receptors [15–17]. In addition to soluble mediators and pattern recognition pathways, direct cell-cell interactions between hepatocytes and macrophages may also contribute to inflammatory amplification during NAFLD progression. However, the molecular mechanisms underlying such contact-dependent signaling remain poorly defined.

Leukocyte immunoglobulin-like receptors (LILRs) in humans and their murine counterparts, paired immunoglobulin-like receptors (PIRs), are paired immune receptors consisting of activating and inhibitory forms. These receptors are primarily expressed on macrophages, monocytes, and B cells, and are essential for orchestrating both innate and adaptive immune responses [18, 19]. As members of the immunoglobulin superfamily, LILRs and PIRs possess Ig-like extracellular domains capable of recognizing a diverse array of ligands, including human leukocyte antigen (HLA) class I molecules [19]. angiopoietin-like proteins (Angptls) [20, 21]. and oligomeric β-amyloid angiopoietin-like proteins [22]. Upon ligand binding, activating receptors (LILRAs, except the soluble LILRA3) signal through the Fc receptor common γ-chain (FcRγ), which contains immunoreceptor tyrosine-based activation motifs (ITAMs) [23]. In contrast, inhibitory receptors (LILRBs), which feature long cytoplasmic tails harboring immunoreceptor tyrosine-based inhibitory motifs (ITIMs), attenuate downstream signaling by recruiting SH2 domain-containing tyrosine phosphatases [24]. LILRs are implicated in various pathological contexts, including cancer [25, 26]. autoimmune disorders, viral infections [27]. and transplant rejection [28–30]. The list of LILR ligands and their functions continues to expand, underscoring their immunological significance.

LILR gene expression is elevated in NAFLD [31–33]. however, their specific ligands and functional roles remain largely undefined. Whether specific ligand-receptor interactions within the hepatic microenvironment directly regulate macrophage activation during NAFLD remains unclear. In this study, we screened murine PIRs and their candidate ligands, identifying PIRA2 as a receptor that recognizes CD1d-a non-classical MHC class I-like molecule-and promotes macrophage activation. Notably, macrophages from *Pira*^−/−^ mice exhibited diminished expression of inflammatory genes. Consistently, *Pira*^−/−^ mice displayed markedly reduced hepatic inflammation and lipid accumulation in a high-fat diet (HFD)-induced NAFLD model.

## Material and methods

### Mice

*Nf1*^−/−^ transgenic mice were purchased from The Jackson Laboratory. *Pira2*^−/−^ transgenic mice were generated by Cyagen (Soochow, China) and genotyped. *Pira2*^*fl/fl*^ mice were generated by GemPharmatech Co., Ltd. (Nanjing, China). *Lyz2-Cre* mice were obtained from Model Organisms (Shanghai, China). CD11b-DTR mice were obtained from GemPharmatech Co., Ltd. Macrophage-specific *Pira2* conditional knockout mice (*Pira2*^*fl/fl*^*Lyz2-Cre*) were obtained by crossing *Pira2*^*fl/fl*^ mice with *Lyz2-Cre* transgenic mice. Littermate *Pira2*^*fl/fl*^ mice lacking Cre recombinase were used as controls. *Rag1*^*−/−*^ and wild-type C57BL/6 J mice were purchased from Charles River Laboratories (Beijing, China). For this study, a cohort of age-matched mice was fed an SD or HFD (60% of energy as fat; D12492; Research Diets, New Brunswick, NJ, USA). All mice were kept in a specific pathogen-free facility with controlled light (06:00 am–06:00 pm) and temperature (22 ± 2 °C) conditions. All animal experiments were conducted according to a protocol approved by Huazhong University of Science and Technology Animal Care and Use Committee (TJH-20220905). Mice were randomly assigned to experimental groups. Investigators were not blinded to group allocation during experiments and outcome assessment.

### Human samples

Blood samples were collected from healthy donors at the Wuhan Blood Center. Liver sections from paraffin-embedded specimens and the clinical data of healthy donors, NAFLD donors, and HCC patients were collected, and H&E staining was used to evaluate the extent of fatty infiltration. The study complied with the Declaration of Helsinki and was approved by the Human Assurance Committee of Tongji Hospital (TJ-IRB20220504).

### Antibodies and tetramers

Sources for antibodies (clone name indicated) against the murine and human targets were listed in Table 1. Isotype control antibodies were purchased from the same source as target-specific antibodies, and goat anti-mouse IgG1 secondary antibodies (A-21121) were obtained from Thermo Fisher. Blocking antibodies for CD1d were purchased from BioLegend (1b1). MHC class I tetramers (H2Db), CD1d tetramers, CD1d tetramer-loaded PBS-57 and CD1d tetramer-loaded OCH were obtained from the NIH Tetramer Core Facility (Emory University, Atlanta, GA).

**Table 1:** Sources for antibodies.

Antibodies	SOURCE	IDENTIFIER
APC anti-mouse F4/80 (clone BM8)	Biolegend	cat#: 123116; RRID: AB_893481
PE anti-mouse CD80 (clone 16-10A1)	Biolegend	cat#: 104708; RRID: AB_313129
FITC anti-mouse CD86 (clone GL-1)	Biolegend	cat#: 105006; RRID: AB_313149
PE/Cyanine7 anti-mouse/human CD11b (clone M1/70)	Biolegend	cat#: 101216; RRID: AB_312799
FITC anti-mouse I-A/I-E (clone M5/114.15.2)	Biolegend	cat#: 107606; RRID: AB_313321
PE anti-mouse PIR-A/B (clone 6C1)	Biolegend	cat#: 144104; RRID: AB_11204242
FITC anti-mouse CD1d (clone 1B1)	Biolegend	cat#: 123508; RRID: AB_1236549
APC anti-human CD14 (clone M5E2)	Biolegend	cat#: 301808; RRID: AB_314190
PE Goat anti-mouse IgG (minimal x-reactivity) Antibody (clone Poly4053)	Biolegend	cat#: 405307; RRID: AB_315010
Rabbit Anti-LILRA2/CD85h	Bioss	cat#: bs-18249R;
Anti-CD14 Mouse mAb	Servicebio	cat#: GB14023;
Phospho-Src Family (Tyr416) Rabbit mAb (clone D49G4)	CST	cat#: 6943 T; RRID: AB_10013641
Src Rabbit mAb (clone 36D10)	CST	cat#: 2109S; RRID: AB_2106059
NF-κB p65 Mouse mAb (clone L8F6)	CST	cat#: 6956S; RRID: AB_10828935
Phospho-NF-κB p65 (Ser536) Rabbit mAb (clone 93H1)	CST	cat#: 3033S; RRID: AB_331284
FcRγ (E6Y1A) Rabbit mAb (clone E6Y1A)	CST	cat#: 78401S;
Anti-Phospho - (Ser/Thr) antibody (clone)	Abcam	cat#: ab117253; RRID: AB_10903259
Anti-beta Actin antibody	Abcam	cat#: ab8227; RRID: AB_2305186

### PIR/Fc or LILRA/Fc fusion proteins

Sequences encoding the extracellular portions of murine PIRA1-6 were amplified by PCR from cDNA isolated from the liver macrophages of immunized C57BL/6 mice. PCR-generated fragments were cloned into the pFUSE-mIgG1-Fc plasmid (InvivoGen). The constructs were transfected into P3X63Ag8.653 cells (ATCC clone CRL-1580), and the expressed protein was purified on a protein G column (GE Healthcare). All cell lines were tested negative for mycoplasma contamination.

### CD1d tetramer and PIRA2/Fc binding assays

pMys-IRES-green fluorescent protein (pMysIG) vectors expressing PIRA1-3 and CD3e were transfected into BWZ.36 reporter cells. The reporter cells were then incubated with 10 µg/ml CD1d, H2Dd tetramers or ctrl IgG for 24 h. β-Galactosidase activity was quantified using a β-galactosidase enzyme assay system (Promega). Maximum β-galactosidase activity was induced by treating reporter cells with PMA (5 ng/ml) + ionomycin (0.5 µg/ml). 3T3 cells transfected with the pMysIG vector expressing PIRA2 were stained with CD1d tetramers and analysed by flow cytometry.

### Quantitative real-time PCR

To assess gene expression in BMDMs, the cells were stimulated with CD1d, and total cellular RNA was extracted using a RNeasy Mini Kit (Qiagen) and reverse transcribed into cDNA (Bio-Rad Laboratories). The expression of genes of interest and of the HPRT control was assessed by PCR using SYBR Green mix (Bio-Rad). Transcript levels of target genes were calculated as the ratio of target gene expression to HPRT expression. Fold changes in target gene expression were analysed by CFX Manager Software (Bio-Rad) using the delta/delta CT method. The sequences for primers were listed in Table 2.

**Table 2:** The sequences for primers.

Species	gene	Sequence of primer
Mus	*Il1b*	Forward: 5′-aag ggc tgc ttc caa acc ttt gac-3‘
		Reverse: 5′-ata ctg cct gcc tga agc tct tgt-3‘
Mus	*Il6*	Forward: 5′-tag tcc ttc cta ccc caa ttt cc-3‘
		Reverse: 5′-ttg gtc ctt agc cac tcc ttc-3‘
Mus	*Tnfα*	Forward: 5′-gcc tct tct cat tcc tgc ttg-3‘
		Reverse: 5′-ggg tct ggg cca tag aac tg-3‘
Mus	*Mcp1*	Forward: 5′-cac tca cct gct gct act cat t-3‘
		Reverse: 5′-tgt ctg gac cca ttc ctt ctt g-3‘
Mus	*inos*	Forward: 5′-gtt ctc agc cca aca ata caa ga-3‘
		Reverse: 5′-gtg gac ggg tcg atg tca c-3‘
Mus	*Cd80*	Forward: 5′-acc ccc aac ata act gag tct-3‘
		Reverse: 5′-ttc caa cca aga gaa gcg agg-3‘
Mus	*Cd86*	Forward: 5′-tgt ttc cgt gga gac gca ag-3‘
		Reverse: 5′-ttg agc ctt tgt aaa tgg gca-3‘
Mus	*Mhcii*	Forward: 5′-agg tga aga cga cat tga gg-3‘
		Reverse: 5′-aac tca gga agc atc cag ac-3‘
Mus	*Cd1d1*	Forward: 5′-aat ctg aag ccc agc aaa aga a-3‘
		Reverse: 5′-tta ctc caa cgg tga gtc tgc-3‘
Mus	*Cd1d2*	Forward: 5′-gtc cca ggg caa gtt gag taa-3‘
		Reverse: 5′-ccc agg gta cat ttc aca gcc-3‘
Mus	*Hprt*	Forward: 5′-agt aca gcc cca aaa tgg tta-3‘
		Reverse: 5′-ctt agg ctt tgt att tgg ctt t-3‘
Homo	*IL1β*	Forward: 5′-atg atg gct tat tac agt ggc aa-3‘
		Reverse: 5′-gtc gga gat tcg tag ctg ga-3‘
Homo	*IL6*	Forward: 5′-cca cac aga cag cca ctc ac-3‘
		Reverse: 5′-agg ttg ttt tct gcc agt gc-3‘
Homo	*TNFα*	Forward: 5′-atc ttc tcg aac ccc gag tga-3‘
		Reverse: 5′-cgg ttc agc cac tgg agc t-3‘
Homo	*iNOS*	Forward: 5′-ttc agt atc aca acc tca gca ag-3‘
		Reverse: 5′-tgg acc tgc aag tta aaa tcc c-3‘
Homo	*HPRT*	Forward: 5′-acc agt caa cag ggg aca taa-3‘
		Reverse: 5′-ctt cgt ggg gtc ctt ttc acc-3‘

### Hepatic macrophages and hepatocytes isolation

Hepatic macrophages and hepatocytes were isolated as described before [34]. In brief, mouse livers were firstly perfused by pre-warmed solution containing 0.05% collagenase type IV dissolved in Ca^2+^/Mg^2+^ HBSS, filtered through a 40μm nylon strainer (Falcon) and were then subjected to 50 g centrifugation. Primary hepatocytes were harvest from cell pellet. To enrich liver mononuclear cells (MNCs), NPCs (nonparenchymal cells) were suspended in HBSS and layered onto a two-layer 25%-50% Percoll gradient (GE Healthcare) and centrifuged. MNCs in the middle layer were collected. For hepatic macrophage enrichment, MNCs were stained with allophycocyanin (APC)-labeled anti-F4/80 antibody, followed by incubation with anti-APC Microbeads. Hepatic macrophages were isolated by passing these labeled MNCs through a magnetic field.

### Hepatocyte-macrophage co-culture assay

Primary hepatocytes were isolated from HFD-fed mice as described above. Bone marrow-derived macrophages (BMDMs) were seeded in 12-well plates and co-cultured with hepatocytes at a 1:1 ratio. In indicated experiments, CD1d-blocking antibody (clone 1B1, BioLegend) or isotype control antibody was added at 10 μg/ml. After 24 h, macrophages were harvested for RNA extraction and quantitative PCR analysis.

### Co-immunoprecipitation assay

BMDMs were stimulated with CD1d tetramers (10 μg/ml) for overnight and lysed in RIPA lysis buffer supplemented with protease and phosphatase inhibitors. Cell lysates were incubated overnight at 4 °C with anti-CD1d antibody or isotype IgG control, followed by incubation with Protein G agarose beads. Immunoprecipitates were washed, resolved by SDS-PAGE, and analysed by immunoblotting with anti-PIRA antibody (Biolegend).

### Blood biochemistry

Mice were under mild pentobarbital anaesthesia. Blood samples were collected from the orbital venous plexus by using heparin lithium-anticoagulant tubes. Blood biochemical indexes were measured with an automated biochemical analyser BS-200 (Mindray, China).

### Glucose Tolerance Test (GTT)

GTT was performed after 20 weeks of HFD treatment. Mice were fasted overnight (12–16 h), weighed and blood glucose was measured by snipping the tail and using a glucose meter (ONE-TOUCH Ultra) before and after intraperitoneal injection of glucose solution or after a oral bolus of glucose solution (2 g/kg body weight). At the end of the test all the tail wounds were cauterized and the mice were provided with food.

### ALT and AST assay

Serum ALT and AST were measured with an ALT Activity Assay kit and AST Activity Assay kit according to the manufacturer’s instructions (Sigma Aldrich).

### RNA-seq and bioinformatic data analysis

Total RNA was extracted from untreated BMDMs or CD1d tetramer treated BMDMs with TRIzol (Invitrogen). Total RNAs (2 μg) were used for stranded RNA sequencing library preparation by means of a Stranded mRNA Library Prep Kit from Illumina (DR08502, Bioyigene) according to the manufacturer’s instructions. The library products corresponding to 200–500 bp were enriched, quantified, and finally sequenced on DNBSEQ-T7. The gene expression profiles of CD1d tetramer stimulated BMDMs and the corresponding control cells were determined by RNA-Seq data analysis (Bioyigene). In brief, raw sequencing data were first filtered by FastQC; low-quality reads were discarded, and adapter sequences were trimmed. After quality filtering, each sample had ~40.4–54.8 million clean reads. Clean reads from each sample were mapped to the Mus musculus GRCm38 reference genome using the hisat2. Significantly differentially expressed transcripts were screened by applying the criteria FC ≥ 2 or ≤ −2 and *P* ≤ 0.05. The RNA-seq data, entitled “Transcriptome RNA-seq analysis for CD1d tetramer-stimulated BMDMs,” were deposited in the Sequence Read Archive (SRA) with BioProject number PRJNA841279. Gene Ontology (GO) analysis of differentially expressed genes was conducted using the GO-seq R package, with a *p*-adjust (FDR) < 0.05 to determine statistically significant enrichment.

### CD1d and LILRA2 correlations

A search of the GEO Profiles database revealed that CD1d and LILRA2 were upregulated in patients with obesity and NAFLD (GEO accession GSE156906) [35]. Expression profiling was performed by high-throughput sequencing of human subcutaneous abdominal adipose tissue. The log2CPM for CD1d and LILRA2 in the metabolically healthy lean group and metabolically unhealthy obese with NAFLD group were analysed.

### Quantification and statistical analysis

Data were presented as mean ± s.e.m. in each group. Comparison of means in two groups was performed using an unpaired two-tailed Student’s *t*-test and those from three groups using one-way ANOVA test (Prism GraphPad, version 6). Correlations between CD1d and LILRA2 levels were assessed by Linear regression. A *p* value less than 0.05 was considered statistic significant. No samples or animals were excluded from the analysis unless technical failure occurred.

## Results

### PIRA2 recognizes CD1d and mediates macrophage activation

**Fig. 1:**
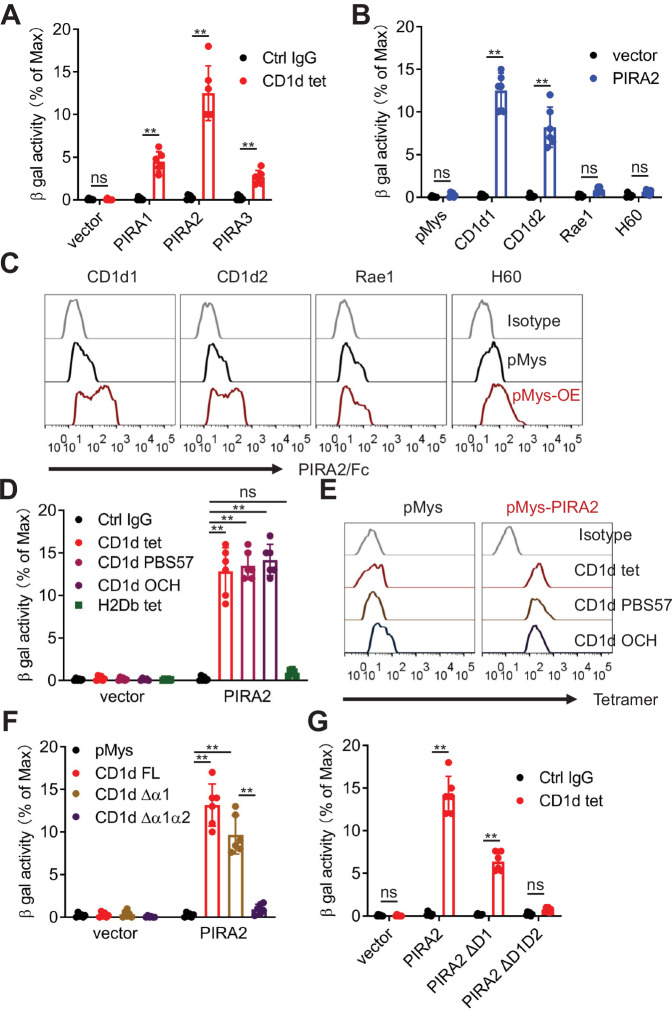
PIRA2 recognized CD1d. **A** PIRA1-3/CD3e chimeric receptor reporters were stimulated with CD1d tetramers or control IgG for 24 h, and then β-galactosidase activity was measured. **B** PIRA2/CD3e chimeric receptor reporters were stimulated with 3T3 cells transfected with pMys-CD1d1, pMys-CD1d2, pMys-Rae1 or pMys-H60 for 24 h, and then β-galactosidase activity was measured. **C** 3T3 cells transfected with pMys empty vector or overexpressed (pMys-OE) CD1d1, CD1d2, Rae1 or H60. Then cells were stained with PIRA2/Fc and examined by a fluorescent-labeled secondary antibody. **D** PIRA1-3/CD3e chimeric receptor reporters were stimulated with CD1d tetramers, PBS-57-loaded CD1d-tetramers or OCH-loaded CD1d tetramers for 24 h, and then β-galactosidase activity was measured. **E** 3T3 cells transfected with pMys-PIRA2 were stained with CD1d tetramers, PBS-57-loaded CD1d-tetramers or OCH-loaded CD1d tetramers and examined. **F** PIRA1-3/CD3e chimeric receptor reporters were stimulated with 3T3 cells transfected with CD1d or CD1d depletion mutants for 24 h, and then β-galactosidase activity was measured. **G** PIRA2 or depletion mutant chimeric receptor reporters were stimulated with CD1d tetramers or ctrl IgG for 24 h, and then β-galactosidase activity was measured. Data were pooled from two independent experiments (*n* = 6). Each symbol represents one individual. All data are the mean ± s.e.m. and were analysed by two-tailed, unpaired Student’s *t* tests (**A**, **B**, **D**, **F**, and **G**). The data are from one of three independent experiments (**C** and **E**).

We previously established a cell-based chimeric receptor screening system and identified PIRA3 as a receptor for allogeneic MHC class I molecules. In this system, chimeric receptors were constructed by fusing the extracellular domains of activating PIRs (PIRA1-3) to the intracellular domain of CD3ε. Upon ligand engagement, these chimeric receptors induce β-galactosidase expression in reporter cells. Using this approach, we screened for potential interactions between PIRs and non-classical MHC class I-like molecules. We found that CD1d tetramers specifically stimulated reporter cells expressing PIRA/CD3ε chimeric receptors, whereas cells expressing CD3ε alone were not activated (Fig. 1A).

To further explore potential ligands for PIRA2, we stimulated PIRA2/CD3ε reporter cells with 3T3 cells expressing various MHC class I-like molecules, including CD1d1, CD1d2, Rae1, and H60. The results showed that PIRA2 selectively recognized CD1d1 and CD1d2, but not Rae1 or H60 (Fig. 1B). Similarly, a PIRA2-Fc fusion protein bound specifically to 3T3 cells expressing CD1d1 and CD1d2, further confirming CD1d as a ligand for PIRA2 (Fig. 1C).

We next examined whether PIRA2 could respond to CD1d loaded with glycolipid antigens. Reporter cells were stimulated with CD1d tetramers alone or loaded with α-GalCer analogs (PBS-57 or OCH). Both unloaded and glycolipid-loaded CD1d tetramers induced β-galactosidase activity, indicating that PIRA2 recognizes CD1d regardless of antigen presentation (Fig. 1D). In contrast, MHC I tetramers (H2Db) did not activate PIRA2 reporters (Fig. 1D). Consistent with this, CD1d tetramers-regardless of antigen loading-bound to 3T3 cells expressing PIRA2, but not to vector-only controls (Fig. 1E), further validating the interaction.

To define the CD1d regions required for binding, we generated CD1d deletion mutants. Reporter assays showed that deleting both α1 and α2 domains abolished PIRA2 stimulation, whereas full-length CD1d and α1-deleted constructs retained activity (Fig. 1F), indicating that the α2 domain is critical for binding. Conversely, deletion of the D1 and D2 domains from PIRA2 eliminated its ability to respond to CD1d tetramers (Fig. 1G), suggesting that these regions are necessary for CD1d recognition.

**Fig. 2:**
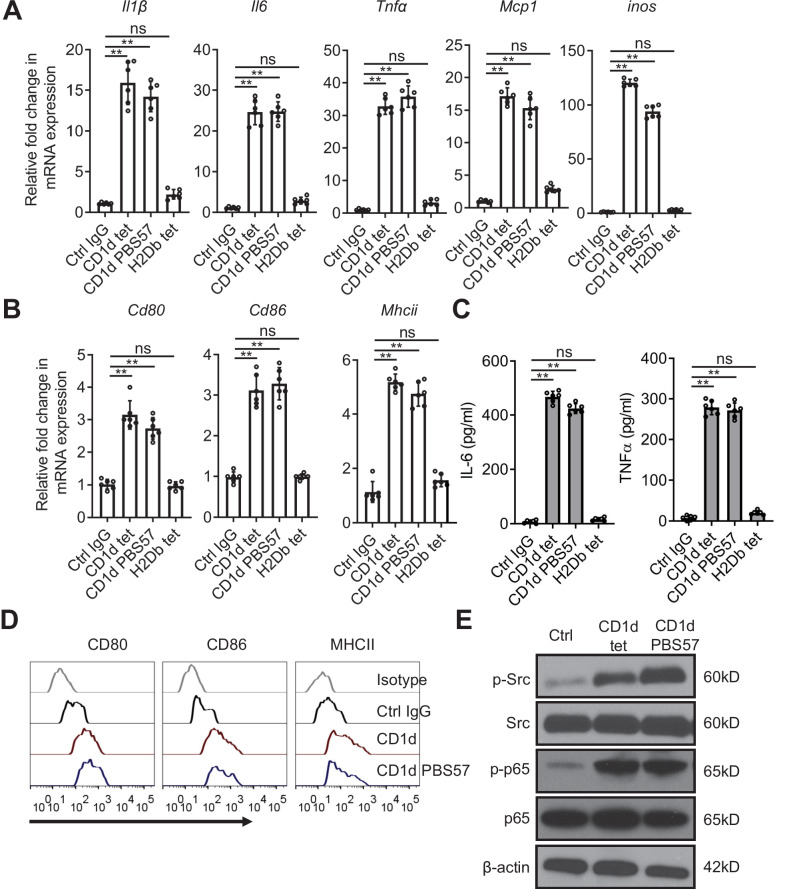
CD1d directly stimulated macrophage activation in vitro. BMDMs were stimulated with CD1d tetramers, PBS-57-loaded CD1d-tetramers, H2Db tetramers or control IgG for 24 h. **A**–**B** qPCR analysis of the mRNA expression of the indicated genes in BMDMs. **C** ELISA analysis of the concentrations of IL-6 and TNFα. **D** Representative FACS histogram of CD80, CD86 and MHCII on BMDMs. **E** Western blot analysis of Src and p65 phosphorylation in BMDMs. Data were pooled from two independent experiments (*n* = 6). Each symbol represents one individual. All data are the mean ± s.e.m. and were analysed by two-tailed, unpaired Student’s *t* tests (**A–C**). The data are from one of three independent experiments (**D**–**E**).

Given the high expression of PIRA receptors in macrophages, we investigated whether CD1d could functionally activate macrophages. Compared to controls and H2Db tetramers, stimulation with CD1d tetramers or α-GalCer-loaded CD1d tetramers significantly upregulated the expression of *Il1β*, *Il6*, *Tnfα*, *Mcp1*, and *inos* in bone marrow-derived macrophages (BMDMs) (Fig. 2A). CD1d also induced the expression of antigen-presentation genes, including *Cd80*, *Cd86*, and *Mhcii* (Fig. 2B). At the protein level, CD1d promoted robust secretion of proinflammatory cytokines (Fig. 2C) and increased surface expression of antigen-presenting molecules (Fig. 2D). RNA-seq analysis further revealed that CD1d stimulation activated immune response pathways and induced gene signatures associated with macrophage activation and polarization (Fig. S1). These data demonstrate that CD1d directly activates macrophages.

Since NF-κB signaling is central to inflammatory gene expression, we assessed p65 phosphorylation. Both CD1d tetramers and glycolipid-loaded tetramers enhanced the phosphorylation of p65 and Src, a tyrosine kinase involved in immune activation (Fig. 2E). Together, these results show that CD1d activates macrophages through NF-κB and inflammatory signaling pathways.

### CD1d mediates macrophage activation through PIRA and the FcR γ chain

**Fig. 3:**
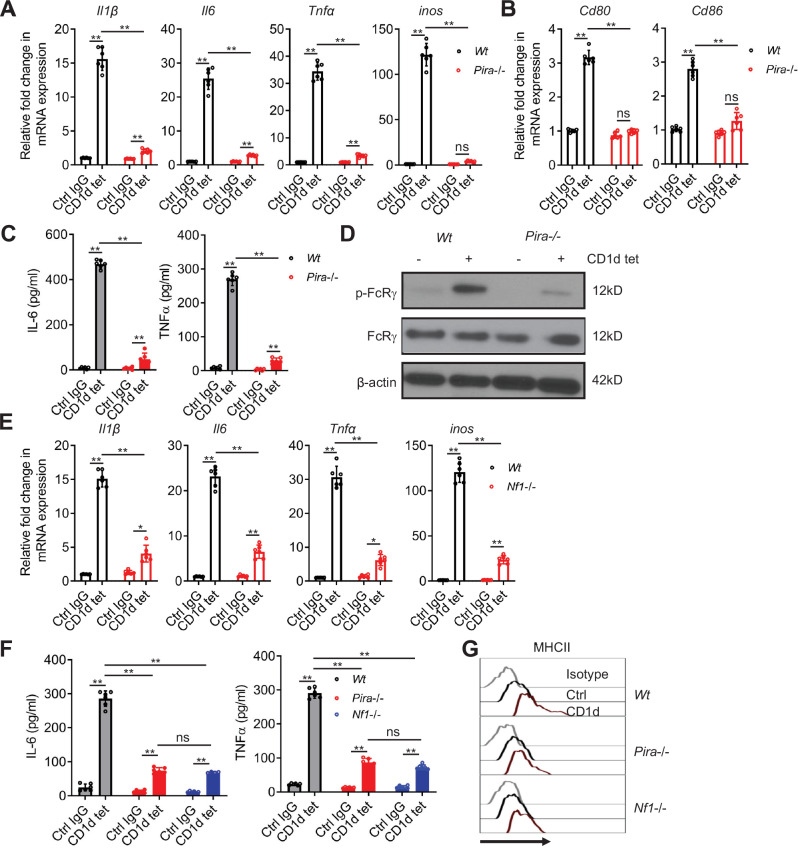
CD1d stimulated macrophage activation through PIRA-FcRγ. **A**–**D** BMDMs from *Wt* and *Pira*^−/−^ mice were stimulated with CD1d tetramers or control IgG for 24 h. **A**–**B** qPCR analysis of the mRNA expression of the indicated genes in BMDMs (**C**) ELISA analysis of the concentrations of IL-6 and TNFα. **D** Western blot analysis of FcRγ phosphorylation in BMDMs. **E**–**F** BMDMs from *Wt* and *Nf1*^−/−^ mice were stimulated with CD1d tetramers or control IgG for 24 h. **E** qPCR analysis of the mRNA expression of the indicated genes in BMDMs. **F** One hundred micrograms of CD1d tetramers or control IgG were injected into *Wt*, *Pira*^−/−^ and *Nf1*^−/−^ mice. Twenty-four hours later, ELISA analysis of the serum concentrations of IL-6 and TNFα was performed. **G** Representative FACS histogram of MHCII on hepatic macrophages. Data were pooled from two independent experiments (*n* = 6). Each symbol represents one individual. All data are the mean ± s.e.m. and were analysed by two-tailed, unpaired Student’s *t* tests (**A**–**C**, **E**–**F**). The data are from one of three independent experiments (**D** and **G**).

Given that PIRA2 recognizes CD1d, we next assessed whether it is required for CD1d-mediated macrophage activation. Compared to wild-type (*Wt*) controls, *Pira*-deficient (*Pira*^*−/−*^) bone marrow-derived macrophages (BMDMs) exhibited significantly reduced expression of inflammatory cytokines following CD1d tetramer stimulation (Fig. 3A), as well as decreased transcription of antigen-presentation genes (Fig. 3B). Correspondingly, levels of IL-6 and TNFα in the culture supernatant of *Pira*^*-/-*^ macrophages were markedly lower than those of *Wt* macrophages upon CD1d stimulation (Fig. 3C). These results indicate that PIRA2 is essential for macrophage activation driven by CD1d.

PIRA2 contains short cytoplasmic tails and a transmembrane domain that associates with the common γ-chain of the Fc receptor (FcRγ). To evaluate whether FcRγ is involved in this pathway, we examined its phosphorylation status after CD1d stimulation. We found that FcRγ was robustly phosphorylated in *Wt* macrophages, but not in *Pira*^*−/−*^ cells (Fig. 3D), consistent with impaired downstream signaling. To further confirm the role of downstream pathways, we assessed macrophages lacking Nf1, which encodes the p50 subunit of the NF-κB pathway. Bone marrow-derived macrophages (BMDMs) from *Nf1*^−/−^ mice produced significantly lower levels of inflammatory cytokines than *Wt* controls after CD1d tetramer stimulation (Fig. 3E, F). Additionally, MHC class II (MHCII) surface expression was markedly diminished in both *Pira*^*−/−*^ and *Nf1*^−/−^ macrophages compared to *Wt* controls (Fig. 3G). Collectively, these findings support a model in which CD1d induces macrophage activation through the PIRA2-FcRγ-NF-κB signaling axis. Given that Nf1 deletion was systemic, the in vivo findings primarily support the involvement of NF-κB signaling rather than establishing macrophage-specific regulation.

### CD1d stimulates macrophage activation in NAFLD mice

**Fig. 4:**
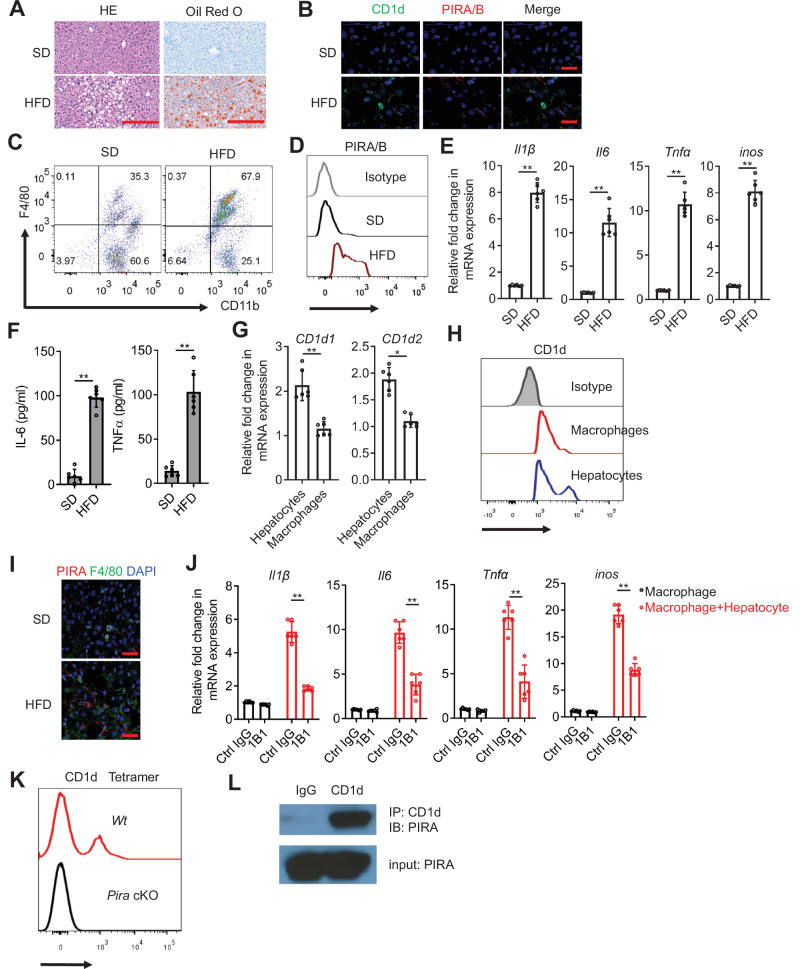
CD1d-PIRA signaling was associated with NAFLD in a mouse model. **A** H&E staining (left panel) and Oil Red O staining (right panel) of liver sections from SD- and HFD-fed mice. Scale bars, 200 µm. **B** Liver sections from SD- and HFD-fed mice were stained for CD1d and PIRA/B. **C** FACS analysis of the hepatic macrophage percentage. **D** FACS analysis of PIRA/B on hepatic macrophages. **E** qPCR analysis of the mRNA expression of the indicated genes in hepatic macrophages. **F** ELISA analysis of the serum concentrations of IL-6 and TNFα. **G** qPCR analysis of the mRNA expression of *Cd1d1* and *Cd1d2* in hepatic macrophages and hepatocytes. **H** FACS analysis of CD1d on hepatic macrophages and hepatocytes. **I** Immunofluorescence staining showing PIRA (red) expression in F4/80⁺ hepatic macrophages (green) in liver sections from SD- and HFD-fed mice. Nuclei were stained with DAPI (blue). Scale bars, 200 µm. **J** Hepatocyte-macrophage co-culture assay showing induction of inflammatory gene expression. **K** CD1d tetramer binding to *Wt* macrophages and Pira2-deficient macrophages. **L** Co-immunoprecipitation showing the interaction between CD1d and PIRA2. Data were pooled from two independent experiments (*n* = 6). Each symbol represents one individual. All data are the mean ± s.e.m. and were analysed by two-tailed, unpaired Student’s *t* tests (**E**–**G**, and **J**). The data are from one of three independent experiments (**A**–**D**, **H**, **I**, **K**, and **L**).

To investigate the physiological relevance of CD1d-PIRA signaling, we used a high-fat diet (HFD) model to induce nonalcoholic fatty liver disease (NAFLD) in wild-type (*Wt*) mice. As expected, HFD-fed mice developed extensive hepatic steatosis, with markedly more lipid droplet accumulation compared to standard diet (SD)-fed controls (Fig. 4A). Immunofluorescence staining of liver sections revealed increased expression of both CD1d and PIRA in HFD-fed mice (Fig. 4B). Additionally, the number of hepatic macrophages was elevated in HFD-fed mice (Fig. 4C). Flow cytometric analysis further showed increased surface PIRA expression on hepatic macrophages from HFD-fed mice (Fig. 4D), consistent with the overall increase observed by immunofluorescence.

We further observed increased mRNA expression of *Il1β, Il6, Tnfα*, and inos in hepatic macrophages isolated from HFD-fed mice (Fig. 4E), along with elevated serum concentrations of IL-6 and TNFα (Fig. 4F), indicating systemic inflammation. Notably, hepatocytes expressed substantially higher levels of *CD1d1* and *CD1d2* mRNA (Fig. 4G) and protein (Fig. 4H) than macrophages. These findings suggest that hepatocyte-derived CD1d may contribute to macrophage activation in the context of NAFLD. To further examine the cellular distribution of PIRA2 in hepatic macrophages, immunofluorescence staining for PIRA and F4/80 was performed. PIRA expression was not uniformly distributed among F4/80⁺ macrophages. Strong PIRA signal was predominantly observed in F4/80^int^ cells, whereas F4/80^hi^ Kupffer-like cells exhibited relatively weaker staining (Fig. 4I), suggesting preferential expression in infiltrating monocyte-derived macrophages.

To directly test this possibility, we performed hepatocyte-macrophage co-culture experiments. Primary hepatocytes isolated from HFD-fed mice induced robust inflammatory gene expression in macrophages (Fig. 4J). Notably, this activation was significantly attenuated by CD1d-blocking antibodies and was abolished when Pira2-deficient macrophages were used, indicating that hepatocyte-derived CD1d contributes to macrophage activation through PIRA2 signaling.

To directly assess receptor-dependent recognition, CD1d tetramer staining was performed. CD1d tetramers showed robust binding to *Wt* macrophages, whereas tetramer binding was markedly reduced in Pira2-deficient macrophages, indicating that PIRA2 is required for CD1d recognition on macrophages (Fig. 4K). Consistently, co-immunoprecipitation assays demonstrated that PIRA2 was specifically detected in CD1d immunoprecipitates but not in IgG controls, confirming a physical interaction between CD1d and PIRA2 (Fig. 4L).

**Fig. 5:**
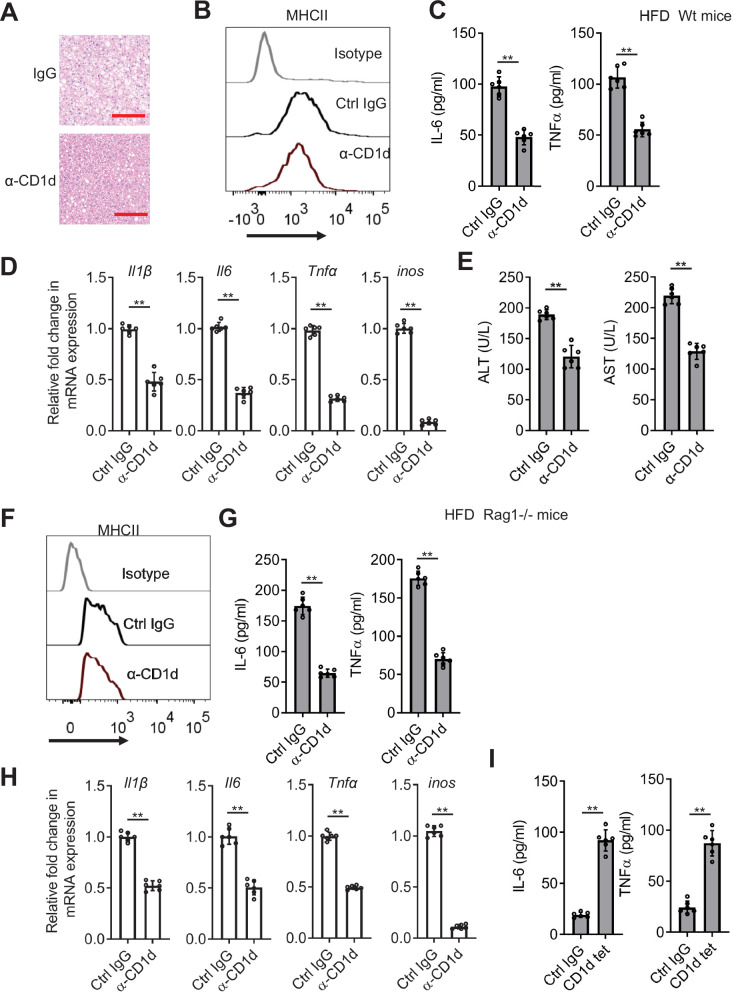
CD1d blockade attenuates macrophage activation and ameliorates NAFLD pathogenesis in vivo. **A**–**E** HFD-fed mice were injected with CD1d blocking or control IgG antibodies. One week later, they were sacrificed and analysed. **A** Representative H&E staining of liver sections. **B** FACS analysis of MHCII on hepatic macrophages. **C** ELISA analysis of the serum concentrations of IL-6 and TNFα. **D** qPCR analysis of the mRNA expression of the indicated genes in BMDMs. **E** Serum ALT and AST levels. **F**–**I** HFD-fed *Rag1*^−/−^ mice were injected with CD1d blocking or control IgG antibodies. One week later, they were sacrificed and analyzed. **F** FACS analysis of MHCII on hepatic macrophages. **G** ELISA analysis of the serum concentrations of IL-6 and TNFα. **H** qPCR analysis of the mRNA expression of the indicated genes in BMDMs. **I** *Rag1*^−/−^ mice were injected with 100 μg of CD1d tetramer or control IgG, and 24 h later, ELISA analysis of the serum concentrations of IL-6 and TNFα was performed. Data were pooled from two independent experiments (*n* = 6). Each symbol represents one individual. All data are the mean ± s.e.m. and were analysed by two-tailed, unpaired Student’s *t* tests (**C**–**E**, **G**–**I**). The data are from one of three independent experiments (**A**, **B**, and **F**).

We next examined the functional role of CD1d in vivo by treating HFD-fed mice with CD1d-blocking antibodies. Histological analysis revealed that CD1d blockade reduced hepatic steatosis compared with control IgG-treated mice (Fig. 5A), indicating an overall improvement in liver pathology. Flow cytometric analysis showed that CD1d blockade led to reduced MHCII surface expression on hepatic macrophages (Fig. 5B), accompanied by decreased serum levels of IL-6 and TNFα (Fig. 5C). In hepatic macrophages, the expression of *Il1β*, *Il6*, *Tnfα*, and inos was also significantly downregulated upon CD1d blockade (Fig. 5D), confirming that CD1d is required for macrophage inflammatory activation in NAFLD. Importantly, CD1d blockade also reduced serum ALT and AST levels (Fig. 5E), further supporting an improvement in liver injury. Consistent with these findings, macrophage depletion using CD11b-DTR mice similarly reduced liver injury and improved metabolic parameters, further supporting a central role of macrophages in NAFLD pathogenesis (Fig. S2).

To exclude the contribution of invariant natural killer T (iNKT) cells, which also recognize CD1d via their invariant TCRs, we performed similar experiments in *Rag1*^−/−^ mice that lack both T and NKT cells. Consistent with findings in *Wt* mice, CD1d blockade in HFD-fed *Rag1*^−/−^ mice reduced MHCII expression on hepatic macrophages (Fig. 5F), lowered serum IL-6 and TNFα levels (Fig. 5G), and diminished *Il1β, Il6, Tnfα*, and inos expression in liver macrophages (Fig. 5H).

Finally, to determine whether CD1d alone could directly activate macrophages, we administered CD1d tetramers into *Rag1*^−/−^ mice. This treatment significantly increased serum IL-6 and TNFα concentrations (Fig. 5I), demonstrating that CD1d is sufficient to directly stimulate macrophages independent of NKT cells.

### PIRA promoted macrophage activation and NAFLD

**Fig. 6:**
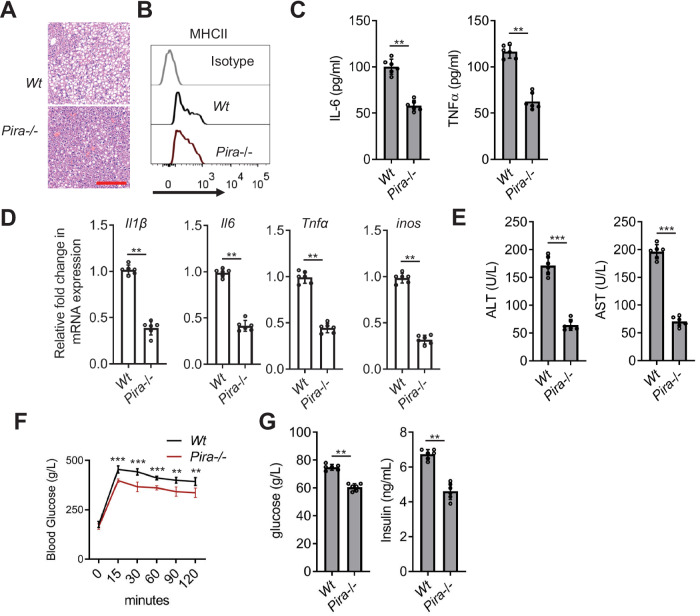
*Pira* deficiency attenuated NAFLD and macrophage activation. **A** H&E staining of liver sections from HFD-fed *Wt* and *Pira*^−/−^ mice. Scale bars, 200 µm. **B** FACS analysis of MHCII on hepatic macrophages from HFD-fed *Wt* and *Pira*^−/−^ mice. **C** ELISA analysis of the serum concentrations of IL-6 and TNFα in HFD-fed *Wt* and *Pira*^−/−^ mice. **D** qPCR analysis of the mRNA expression of the indicated genes in hepatic macrophages from HFD-fed *Wt* and *Pira*^−/−^ mice. **E** ALT and AST of HFD-fed *Wt* and *Pira*^−/−^ mice. **F** GTTs of HFD-fed *Wt* and *Pira*^−/−^ mice. **G** Serum glucose and insulin levels in HFD-fed *Wt* and *Pira*^−/−^ mice. Data were pooled from two independent experiments (*n* = 6). Each symbol represents one individual. All data are the mean ± s.e.m. and were analysed by two-tailed, unpaired Student’s *t* tests (**C**–**G**). The data are from one of three independent experiments (**A**–**B**).

Building on our previous findings that CD1d expression is elevated and contributes to macrophage activation in NAFLD, we investigated the role of PIRA in disease progression. Genetic deletion of Pira significantly reduced hepatic lipid droplet accumulation in HFD-fed mice (Fig. 6A), while metabolic parameters remained comparable between wild-type (*Wt*) and *Pira*^−/−^ mice (Fig. S3). In line with this, Pira deficiency partially decreased MHCII expression on hepatic macrophages (Fig. 6B) and led to reduced serum levels of IL-6 and TNFα (Fig. 6C). Furthermore, mRNA expression of *Il1β, Il6, Tnfα*, and inos in hepatic macrophages was significantly attenuated in *Pira*^−/−^ mice (Fig. 6D), reinforcing the requirement of PIRA in mediating macrophage-driven inflammation.

Given the association between inflammation, NAFLD, and obesity, we further assessed serum levels of liver injury markers aspartate aminotransferase (AST) and alanine aminotransferase (ALT). Both ALT and AST were significantly lower in *Pira*^−/−^ mice fed an HFD (Fig. 6E). Glucose tolerance tests (GTTs) revealed that Pira deficiency conferred protection against glucose intolerance in HFD-fed mice (Fig. 6F), with corresponding reductions in serum glucose and insulin levels (Fig. 6G). Together, these findings suggest that PIRA is a critical mediator of metabolic inflammation and NAFLD progression.

**Fig. 7:**
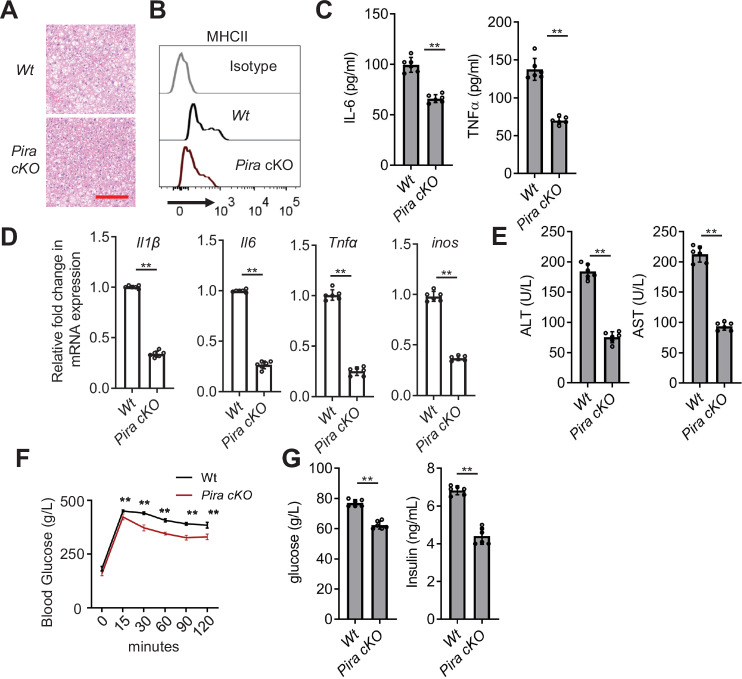
Macrophage-specific deletion of *Pira2* attenuated macrophage activation and NAFLD progression. **A** H&E staining of liver sections from HFD-fed *Wt* and *Pira2*^*fl/fl*^*Lyz2-Cre* mice. Scale bars, 200 µm. **B** FACS analysis of MHCII on hepatic macrophages from HFD-fed *Wt* and *Pira2* cKO mice. **C** ELISA analysis of serum IL-6 and TNFα in HFD-fed *Wt* and *Pira2* cKO mice. **D** qPCR analysis of *Il1β*, *Il6*, *Tnfα*, and *inos* in hepatic macrophages from HFD-fed *Wt* and *Pira2* cKO mice. **E** Serum ALT and AST levels of HFD-fed *Wt* and *Pira2* cKO mice. **F** GTT analysis of HFD-fed *Wt* and *Pira2* cKO mice. **G** Serum glucose and insulin levels in HFD-fed *Wt* and *Pira2* cKO mice. Data were pooled from two independent experiments (*n* = 6). Each symbol represents one individual. All data are the mean ± s.e.m. and were analysed by two-tailed, unpaired Student’s *t* tests (**C**–**G**). The data are from one of three independent experiments (**A**–**B**).

To further determine whether the protective phenotype observed in *Pira*^−/−^ mice was specifically attributable to macrophage-intrinsic PIRA2 signaling, we next examined macrophage-specific *Pira2* conditional knockout mice (*Pira2*^*fl/fl*^*Lyz2-Cre, Pira2* cKO). Similar to global Pira deficiency, macrophage-specific deletion of *Pira2* significantly reduced MHCII expression on hepatic macrophages under HFD conditions (Fig. 7A–C). In addition, proinflammatory cytokine expression, including *Il1β*, *Il6*, *Tnfα*, and inos, was markedly decreased in hepatic macrophages from *Pira2* cKO mice (Fig. 7D). Serum ALT, AST, glucose, and insulin levels were also reduced compared with littermate controls (Fig. 7E–G), indicating ameliorated hepatic inflammation and metabolic dysfunction. These findings confirm that PIRA2 promotes macrophage activation and NAFLD progression in a macrophage-intrinsic manner.

**Fig. 8:**
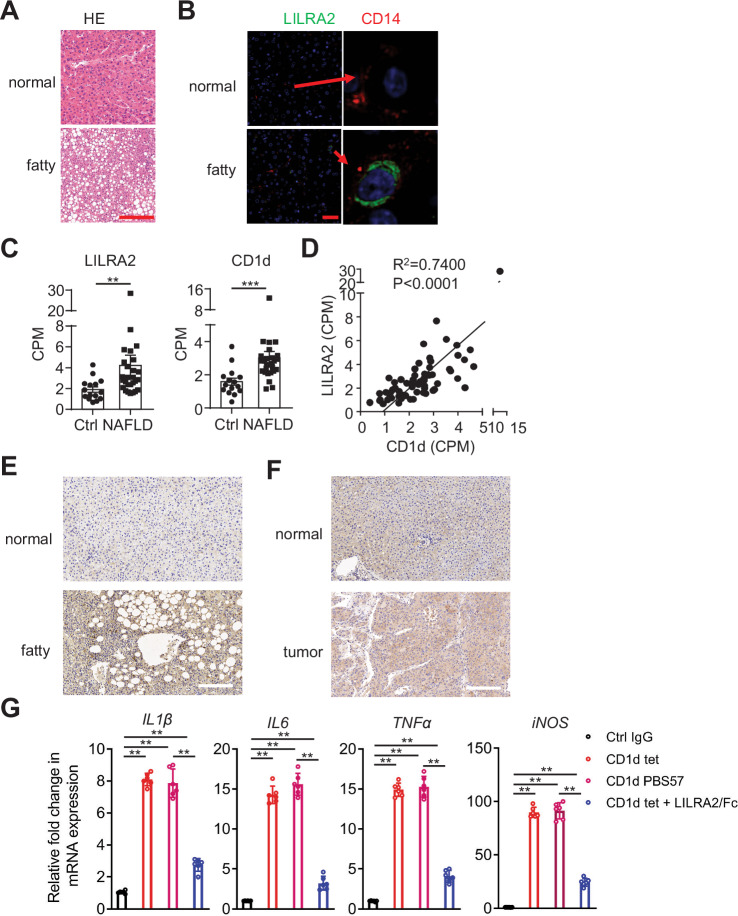
CD1d-LILRA2 axis is elevated in human NAFLD and promotes macrophage activation. **A** H&E staining of liver sections from healthy and NAFLD individuals. Scale bars, 200 µm. **B** Liver sections from healthy and NAFLD individuals were stained for CD1d and LILRA2. Scale bars, 400 µm. **C**–**D** Analysis of CD1d and LILRA2 levels and their relationship in healthy and NAFLD individuals (healthy control *n* = 23, NAFLD *n* = 27). **E** Immunohistochemical analysis of CD1d in liver sections from healthy and NAFLD individuals. Scale bars, 200 µm. **F** Immunohistochemical analysis of CD1d in liver sections from healthy and HCC individuals. Scale bars, 200 µm. **G** Human CD14^+^ PBMC-derived macrophages were stimulated with CD1d tetramers or control IgG for 24 h. qPCR analysis of the mRNA expression of the indicated genes. Data were pooled from two independent experiments (*n* = 6). Each symbol represents one individual. All data are the mean ± s.e.m. and were analysed by two-tailed, unpaired Student’s *t* tests (**G**). The data are from one of three independent experiments (**A**, **B**, **E**, and **F**).

To assess the clinical relevance of our results, we examined liver tissues from individuals diagnosed with NAFLD. In steatotic liver sections with visible lipid droplet accumulation (Fig. 8A), immunofluorescence revealed strong colocalization of LILRA2 with CD14⁺ macrophages and markedly higher expression of LILRA2 compared to normal liver tissues (Fig. 8B). These findings were supported by external datasets showing increased CD1d and LILRA2 expression in obese and NAFLD patients (Fig. 8C) [35]. The relative expression levels of both markers were consistently elevated (Fig. 8D) [35]. Notably, both CD1d and LILRA2 expression levels positively correlated with NAFLD disease severity in these datasets, suggesting that activation of the CD1d-PIRA2/LILRA2 axis may increase during disease progression. Histological analysis further confirmed that CD1d expression was upregulated in NAFLD liver sections with prominent steatosis (Fig. 8E) as well as in hepatocellular carcinoma (HCC) tissues (Fig. 8F). These observations further support the notion that CD1d-mediated signaling may be progressively enhanced during chronic liver inflammation and disease progression.

To functionally validate these findings, we isolated CD14⁺ monocytes from human peripheral blood mononuclear cells (PBMCs) and differentiated them into macrophages using human CSF. Treatment with CD1d tetramers-either unloaded or loaded with glycolipid antigens-robustly induced expression of *IL1β, IL6, TNFα*, and *iNOS* mRNA (Fig. 8G). To investigate whether this effect was mediated by LILRA2, we generated a LILRA2/Fc fusion protein capable of competitively binding CD1d. The fusion protein significantly inhibited CD1d-induced inflammatory gene expression in macrophages (Fig. 8G), demonstrating that CD1d upregulation promotes macrophage activation through LILRA2 in human NAFLD.

## Discussion

Nonalcoholic fatty liver disease (NAFLD) is characterized by sterile inflammation and activation of innate immune cells, including macrophages [1, 36]. While targeting downstream inflammatory pathways can reduce cytokine production, such strategies often increase susceptibility to infection [37, 38]. Therefore, identifying upstream regulators that initiate innate immune activation is a more promising therapeutic approach. In this study, we identified PIRA2 as a novel receptor that recognizes CD1d, regardless of whether it is loaded with antigen. Upon binding, PIRA2 transduces an activation signal through phosphorylation of the ITAM-bearing adapter molecule FcRγ. In vitro experiments demonstrated that CD1d directly activated macrophages and promoted robust inflammatory responses. This activation was dependent on PIRA, as Pira-deficient macrophages failed to produce cytokines in response to CD1d stimulation. Consistently, both CD1d and PIRA expression were elevated in the livers of HFD-fed mice, and blocking their interaction significantly attenuated macrophage activation. Systemic deletion of Pira not only reduced hepatic lipid accumulation but also improved glucose metabolism. Notably, macrophage-specific deletion of *Pira2* recapitulated these protective effects, demonstrating that PIRA2 functions in a macrophage-intrinsic manner in NAFLD. Furthermore, in individuals with NAFLD, CD1d and its human ortholog LILRA2 were both upregulated and contributed to macrophage-driven inflammation.

In the context of NAFLD and obesity, hepatic macrophages can be activated by various small molecules through pattern recognition receptors (PRRs) [39]. Over the past few decades, PRRs and their soluble ligands-including damage-associated molecular patterns (DAMPs) and metabolic intermediates-have been extensively studied [15]. However, despite the clear link between inflammation and NAFLD progression, no anti-inflammatory strategy has been approved for clinical treatment of NAFLD [38]. One likely explanation is that PRRs and their downstream signaling pathways are also essential for antiviral defense and immune surveillance. Thus, identifying targets more specifically associated with NAFLD pathogenesis may offer safer and more effective therapeutic options. During NAFLD, lipid accumulation induces CD1d expression in hepatocytes [40]. Through a cell-based chimeric receptor screening approach, we identified CD1d as a surface ligand of PIRA that triggers macrophage inflammatory activation. As a non-classical MHC class I-like molecule, CD1d binds both self- and microbial glycolipids and presents them to invariant natural killer T (iNKT) cells. Its expression is known to be upregulated in response to lipid stimuli [41]. In our study, CD1d expression was elevated in fatty liver tissues and positively correlated with PIRA2 or LILRA2 expression in both the mouse model and human NAFLD samples, consistent with existing literature. These observations also suggest that activation of the CD1d-PIRA2/LILRA2 axis may increase progressively during NAFLD development as lipid accumulation and inflammatory signaling intensify. Importantly, our data show that CD1d can stimulate macrophage activation independently of NKT cells. In vitro, CD1d directly induced PIRA-dependent proinflammatory cytokine production by macrophages. In *Rag1*^−/−^ mice lacking T and NKT cells, CD1d still triggered inflammatory responses, and CD1d blockade significantly attenuated macrophage activation. Together, these findings indicate that CD1d functions not only as an antigen-presenting molecule for iNKT cells but also as a surface ligand that directly activates macrophages via the PIRA pathway, contributing to inflammation in NAFLD.

PIRA/LILRA receptors are known to recognize self and non-self classical MHC class I or HLA class I molecules [19, 42]. However, the ligands for certain PIRAs, such as PIRA2, have remained unidentified. In this study, we discovered that PIRA2/LILRA2 binds to CD1d. Unlike the T-cell receptor (TCR) on invariant NKT cells, which recognizes CD1d only when it is loaded with glycolipid antigens, PIRA2 is capable of binding both native CD1d and antigen-loaded CD1d. Importantly, Pira deficiency attenuated the macrophage inflammatory response induced by CD1d, consistent with our observation that the LILRA2/Fc fusion protein impaired human macrophage activation in the presence of CD1d tetramers. Interestingly, LILRB2 has previously been reported to bind native CD1d and suppress lipid antigen presentation [31]. In contrast, our results show that LILRA2/PIRA2 engagement by CD1d triggers FcRγ-dependent ITAM signaling and promotes macrophage activation. This may be explained by the high degree of structural similarity among these receptors, despite differences in their cytoplasmic tail lengths and sequences. CD1d is a heterodimer consisting of a CD1d heavy chain and β2-microglobulin (β2m). Previous studies have demonstrated that CD1d interacts with LILRB1 through its N-terminal Ig-like domains D1 and D2 [18, 31]. However, our findings suggest that the interaction between CD1d and PIRA2 is largely independent of β2m, as MHC class I tetramers failed to stimulate PIRA2-expressing reporter cells, whereas CD1d tetramers succeeded. Further mapping experiments indicated that the α1 and α2 domains of CD1d are involved in its interaction with PIRA2. Upon engagement, CD1d triggers phosphorylation of tyrosine residues within the immunoreceptor tyrosine-based activation motif (ITAM) of FcRγ, leading to downstream signaling and proinflammatory activation. This signaling cascade was disrupted in macrophages lacking PIRA, confirming its functional importance in mediating CD1d-induced inflammation.

Notably, aberrant expression of LILR family members has been implicated in various human autoimmune diseases and immune tolerance [43]. For example, significantly lower levels of the inhibitory receptor LILRB1 have been reported in patients with systemic lupus erythematosus (SLE) compared to healthy individuals [44]. LILRB2 has been shown to modulate inflammatory cell activity by recognizing different forms of HLA-B27 in inflammatory arthritis [45]. and contributes to maternal-fetal tolerance by regulating the function of myeloid-derived suppressor cells (MDSCs) [46]. Moreover, serum levels of LILRA3 correlate with disease activity in patients with rheumatoid arthritis (RA) [47]. In this study, we demonstrate that LILRA2 is elevated in individuals with NAFLD and promotes macrophage-driven production of inflammatory cytokines. In a mouse model, Pira deficiency markedly reduced lipid droplet accumulation in the livers of HFD-fed mice. As surface membrane proteins, PIRAs offer unique advantages as therapeutic targets-unlike intracellular nuclear receptors such as PPARs, lanifibranor, or farnesoid X receptor (FXR), PIRAs can be more readily targeted with blocking antibodies or fusion proteins. Additionally, PIRAs act as upstream sensors that initiate innate immune activation, providing a clearer therapeutic window than complex downstream signaling pathways [38].

Given the role of PIRA2/LILRA2 in lipid-induced macrophage activation, this receptor-ligand complex represents a promising therapeutic target in NAFLD. It is capable of responding to both native CD1d and CD1d loaded with lipid antigens-molecules closely tied to lipid metabolism and adipocyte biology in obesity and NAFLD. Through FcRγ-dependent signaling, PIRA2/LILRA2 mediates inflammatory responses in macrophages. Together, our findings shed light on a novel molecular mechanism that drives inflammation in obesity, NAFLD, and nonalcoholic steatohepatitis (NASH), and underscore PIRA2/LILRA2 as a potential upstream immunomodulatory target.

## Conclusions

Macrophages play a central role in liver inflammation associated with nonalcoholic fatty liver disease (NAFLD) and its progressive form, nonalcoholic steatohepatitis (NASH). However, the upstream signals that trigger macrophage activation in this context remain incompletely understood. While increased expression of leukocyte immunoglobulin-like receptors (LILRs) has been reported in NAFLD, the specific ligands and functional consequences of LILR activation have not been fully elucidated.

In this study, we identify CD1d-a non-classical MHC class I molecule-as a novel ligand for PIRA2 during NAFLD. We demonstrate that CD1d binds to PIRA2 via its α1 and α2 domains, while PIRA2 interacts through its D1 and D2 domains. This interaction initiates phosphorylation of the immunoreceptor tyrosine-based activation motif (ITAM) within FcRγ, triggering downstream inflammatory signaling in macrophages.

To our knowledge, this is the first study to characterize CD1d as a ligand for PIRA2 on macrophages. Moreover, we show that the PIRA2/LILRA2 axis actively contributes to macrophage-mediated inflammation through FcRγ-dependent signaling. These findings uncover a novel molecular pathway that may initiate inflammation in obesity, NAFLD, and NASH, and highlight PIRA2/LILRA2 as a promising therapeutic target for modulating innate immune responses in metabolic liver diseases.

## Supplementary information

Figure S1-S3

unprocessed wb images

## Data Availability

All unique/stable reagents used or generated in this study will be made available on request. The accession number for RNA-seq data reported in this paper is BIOPROJECT: PRJNA841279.
