# Supplementary material for: CD1d functions as a ligand for PIRA2 to drive macrophage activation in nonalcoholic fatty liver disease
Source: Cell Death Dis. 2026 Apr 27;17(1):558. doi: 10.1038/s41419-026-08789-9 (PMC13253836; doi:10.1038/s41419-026-08789-9)
Supplement: Supplementary file 1 — Figure S1-S3 [file 41419_2026_8789_MOESM1_ESM.docx]

**Supplementary Figures**

**
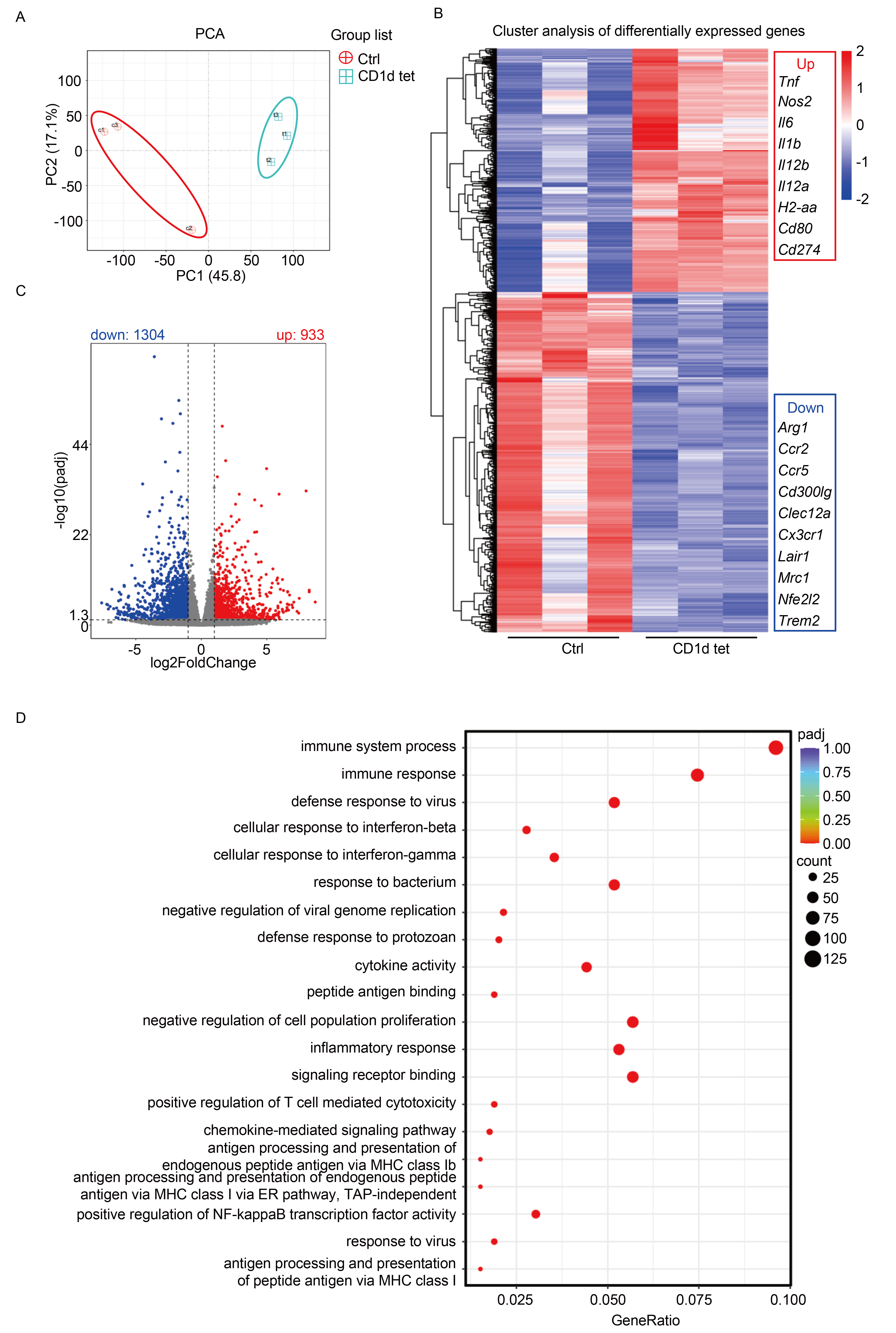
Figure S1**

**Figure S1. CD1d tetramer induced transcriptome associated with immune response in BMDMs.**

BMDMs were stimulated with CD1d tetramers, or control IgG for 24 h. Total RNA was extracted for RNA-seq and bioinformatic data analysis.

(A) Principal component analysis (PCA) plot from the full transcriptome of untreated BMDMs (Ctrl) and CD1d tetramer-treated BMDMs (CD1d tet) for 24 h.

(B) Clustered heatmap of significantly differentially expressed mRNAs in control and CD1d tetramer-treated BMDMs. Some representative upregulated (red matrix) and down-regulated (blue matrix) genes related to macrophage activation and polarization are shown.

(C) Volcano plots indicating the significance of upregulated genes (red) and down-regulated genes (blue) between untreated and CD1d tetramer-treated BMDMs with numbers of differentially expressed genes.

(D) Scatter plot showing the Top 20 Gene Ontology (GO) enrichment results in the upregulated genes. The dot size indicates the relative number of differentially expressed genes contained in the GO terms, and the shade of the dots indicates the extent of the enrichment.


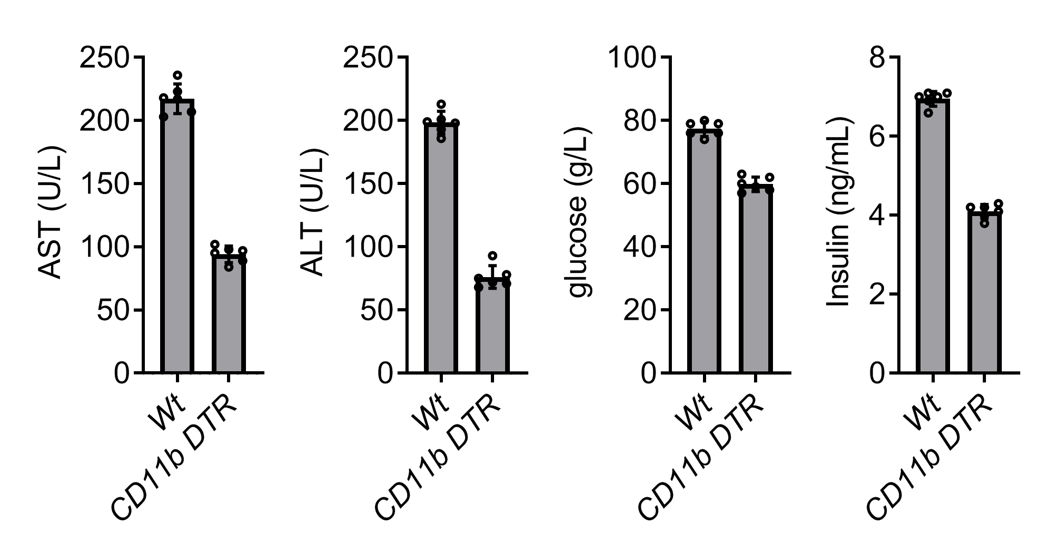
**Figure S2**

**Figure S2. Macrophage depletion attenuates liver injury and improves metabolic parameters in HFD-fed mice.** CD11b-DTR mice and *Wt* controls were subjected to HFD feeding. Macrophages were depleted in CD11b-DTR mice by intraperitoneal injection of diphtheria toxin. Serum AST and ALT levels were measured to assess liver injury. Blood glucose and serum insulin levels were analysed to evaluate metabolic status. Data are presented as mean ± s.e.m. (n=6 mice per group). Each symbol represents one individual mouse. Statistical significance was determined by two-tailed unpaired Student’s t test.


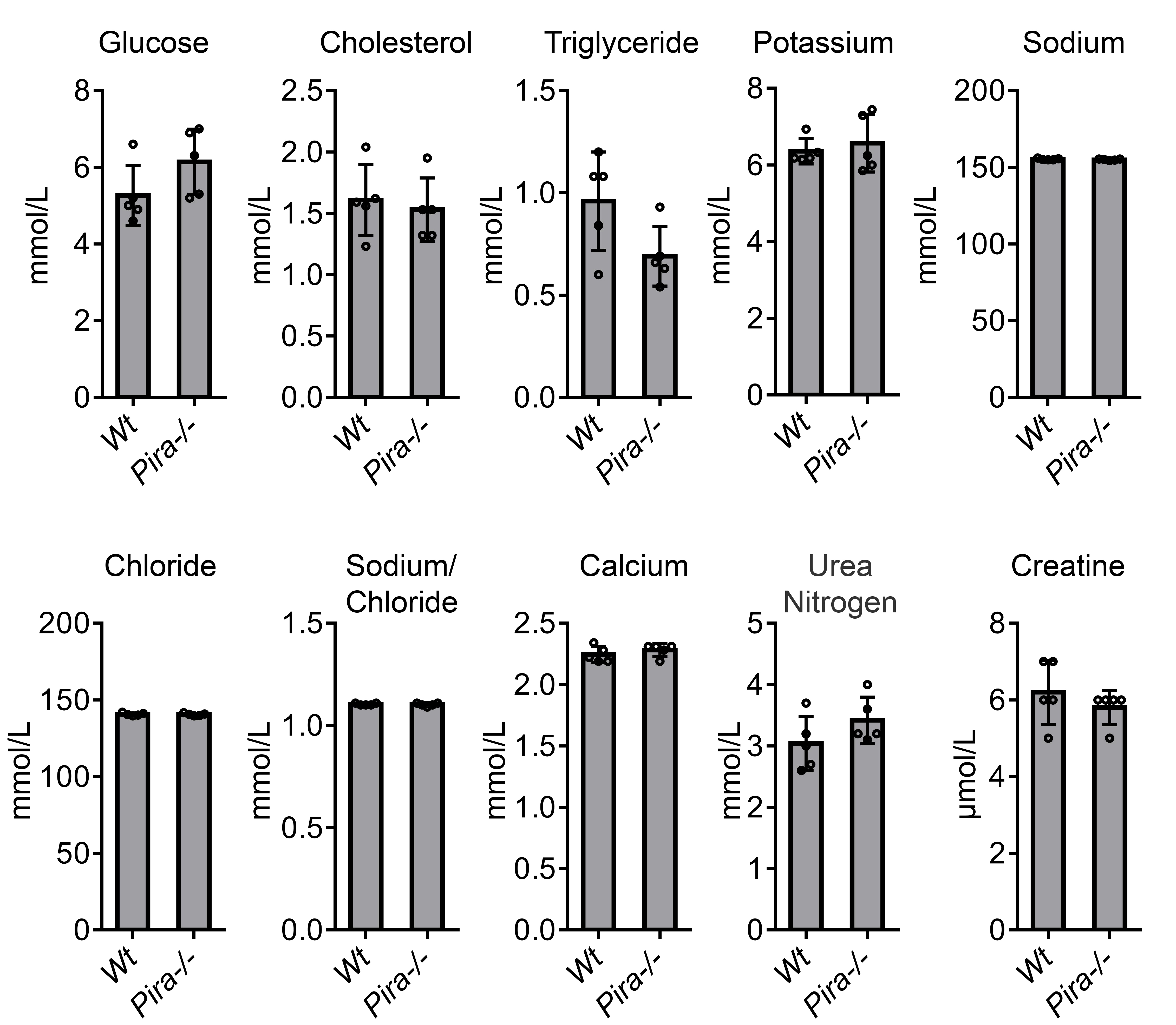
**Figure S3**

**Figure S3. Metabolic panels of *Wt* and *Pira*^-/-^ mice**.

Blood samples were collected from *Wt* and *Pira*^-/-^ mice at 6 weeks. Blood biochemical levels were analysed by an automatic biochemical analyser.

Data were pooled from two independent experiments (n=5). Each symbol represents one individual. All data are the mean ± s.e.m. and were analysed by two-tailed, unpaired Student’s t tests.
