# Supplementary figures and images for: CD1d functions as a ligand for PIRA2 to drive macrophage activation in nonalcoholic fatty liver disease
Source: Cell Death Dis. 2026 Apr 27;17(1):558. doi: 10.1038/s41419-026-08789-9 (PMC13253836; doi:10.1038/s41419-026-08789-9)

Unprocessed original images of gels and western blots

Fig. 2E

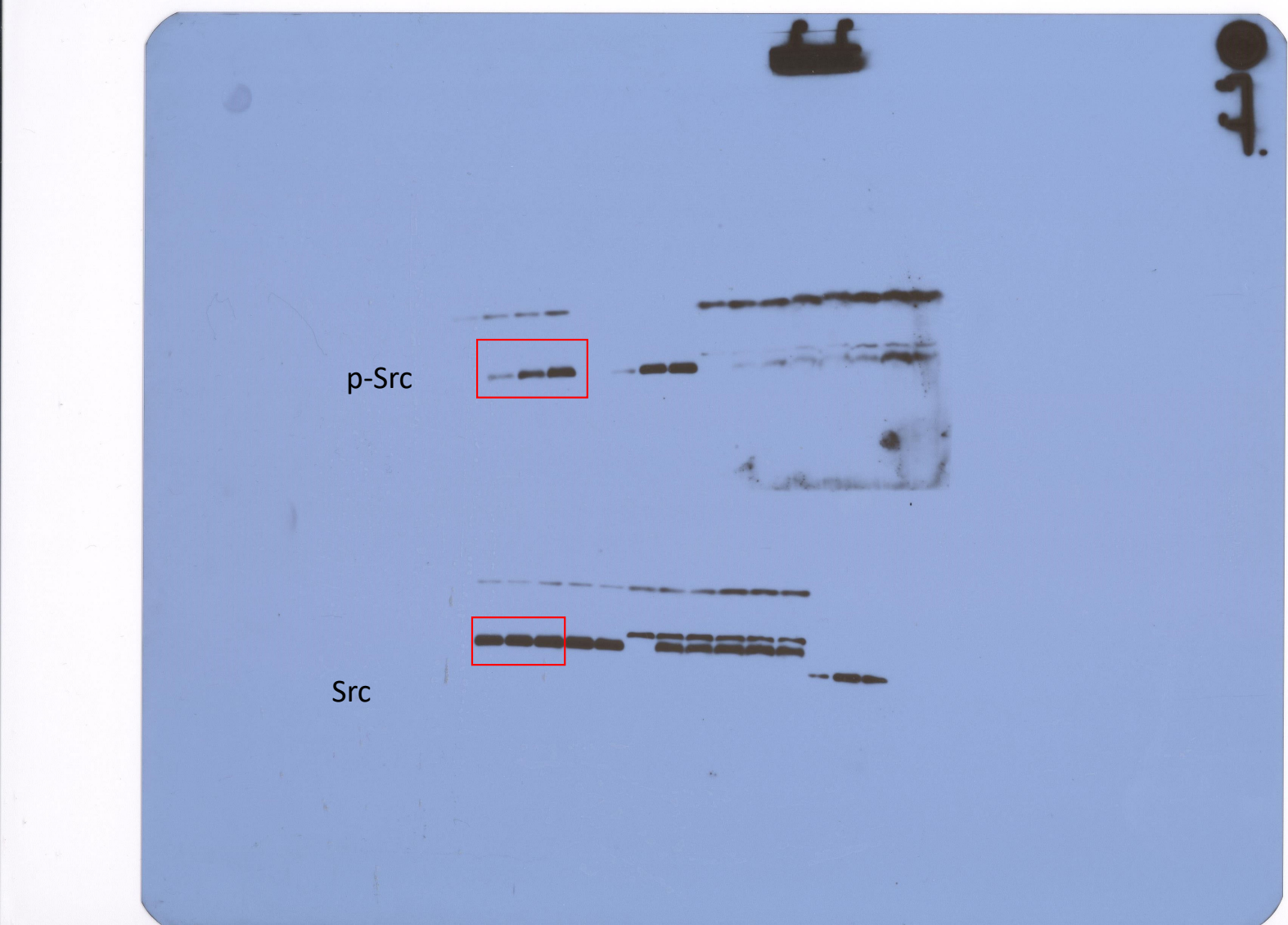

Fig. 2E

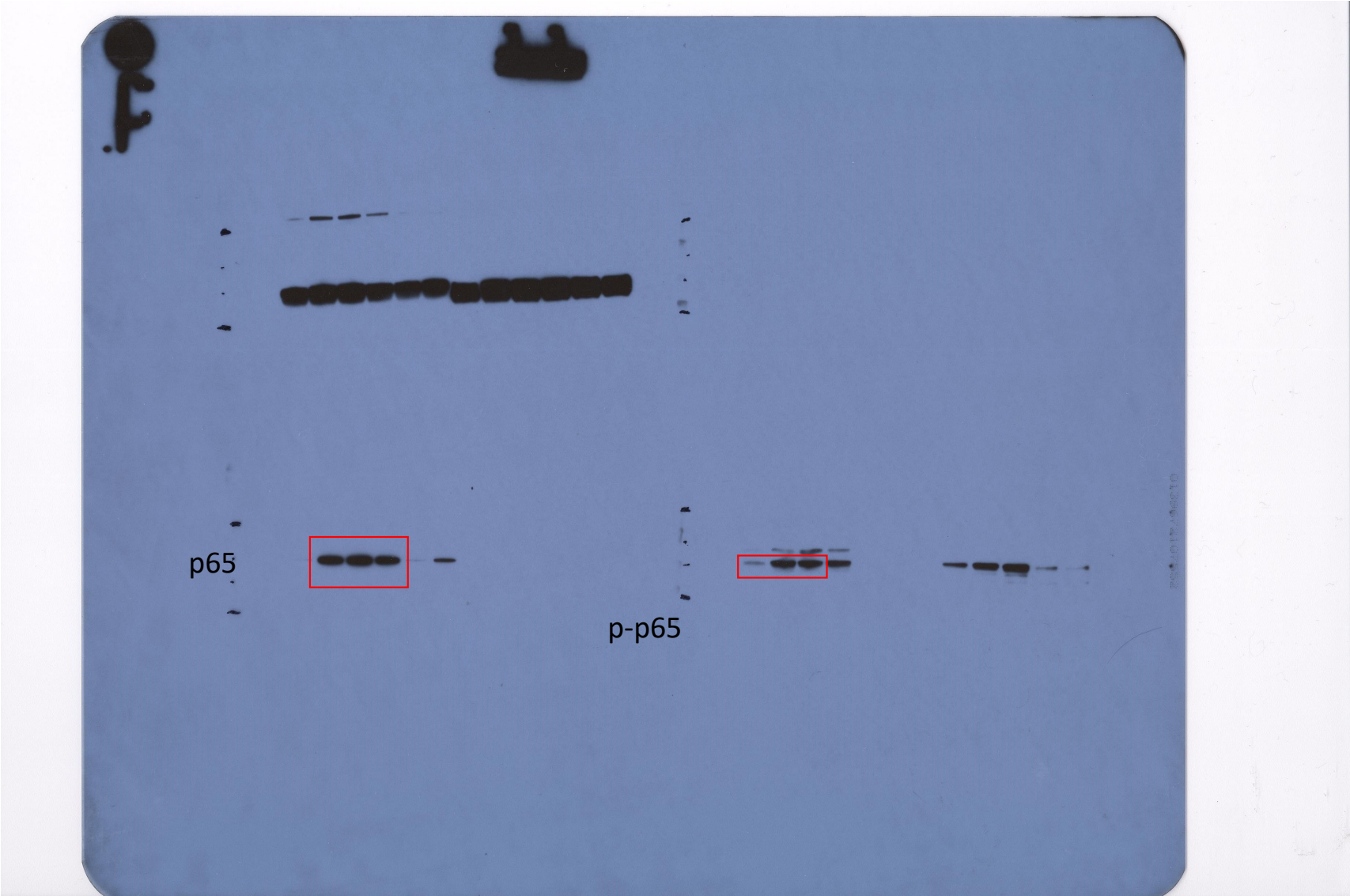

Fig. 2E

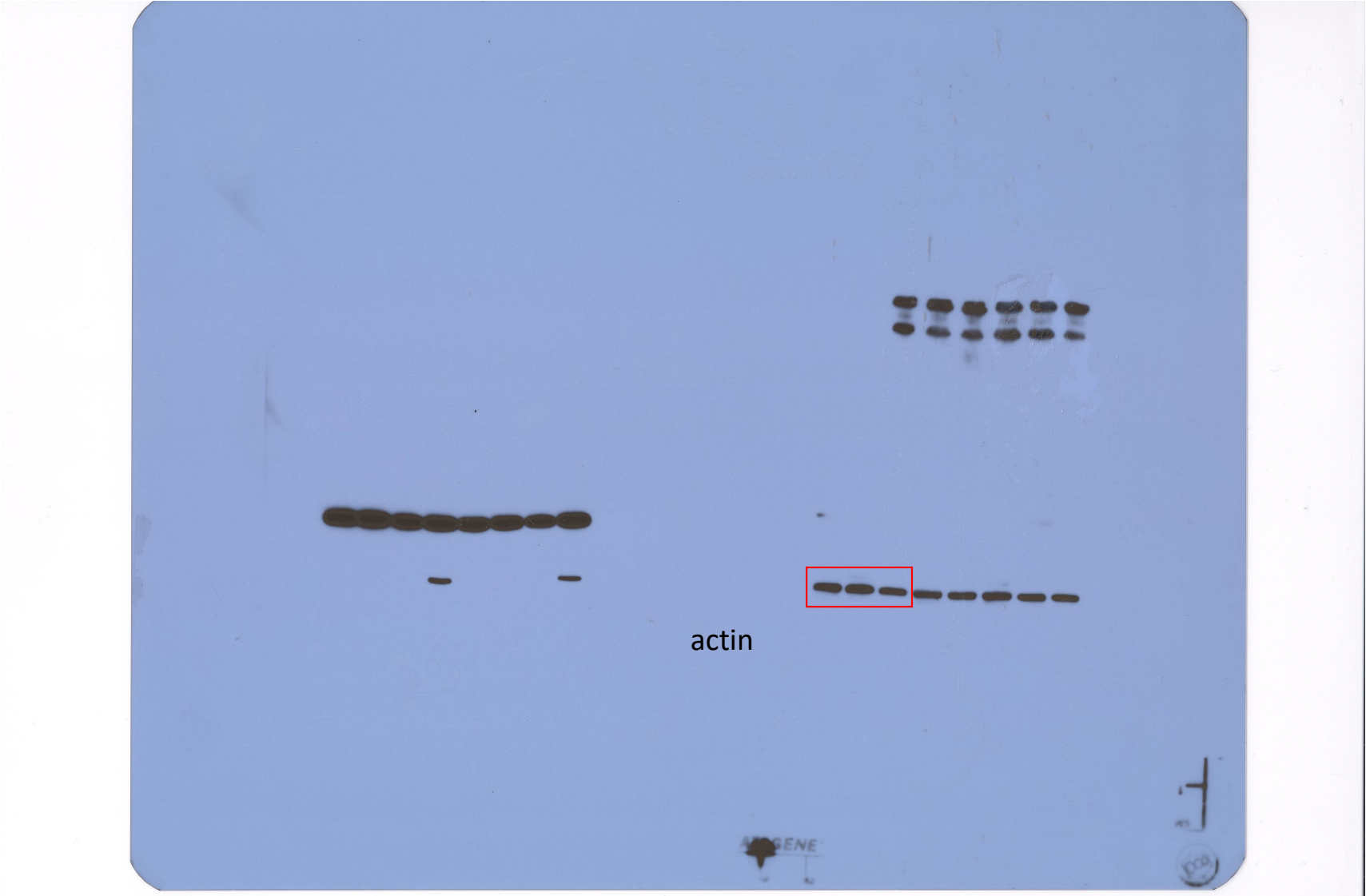

Fig. 3D

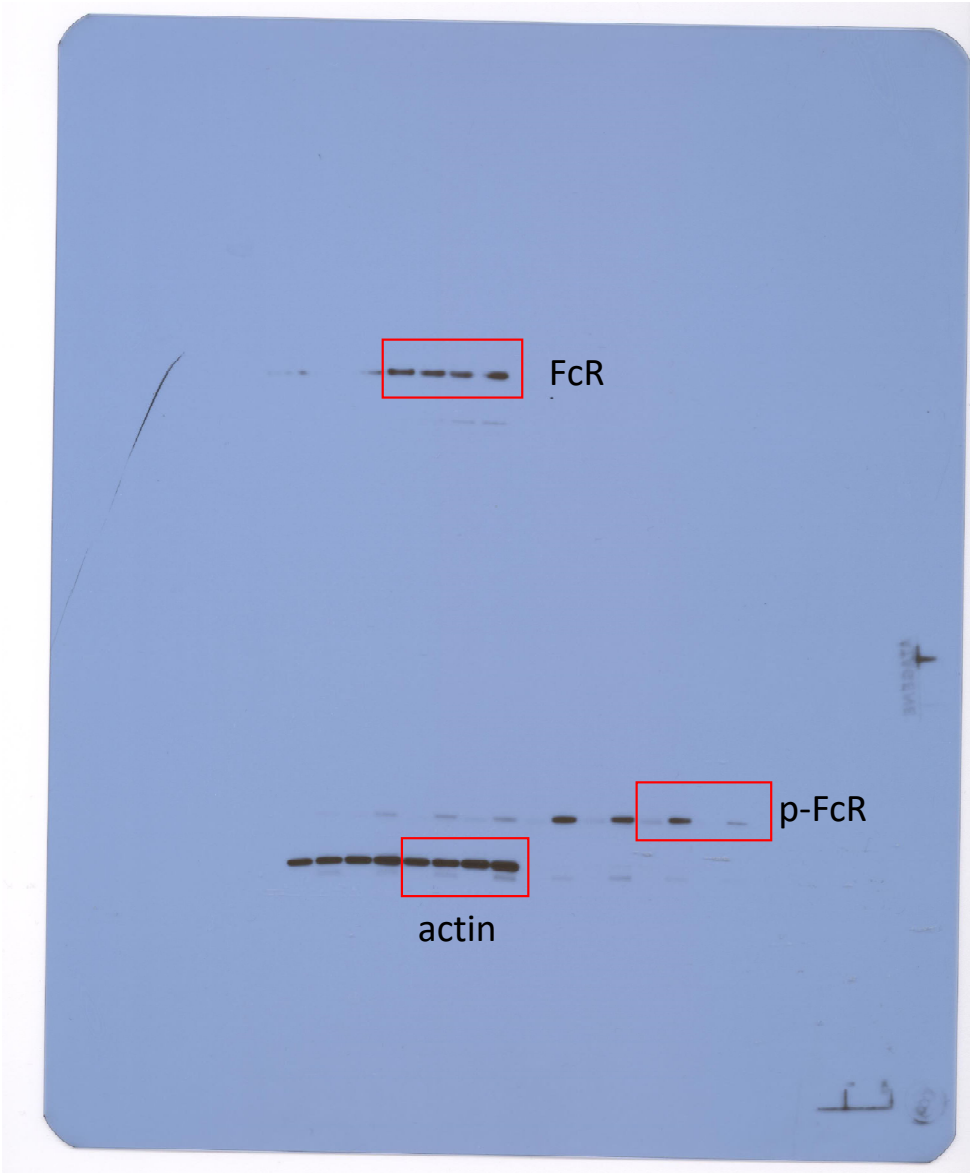

Fig. 4L

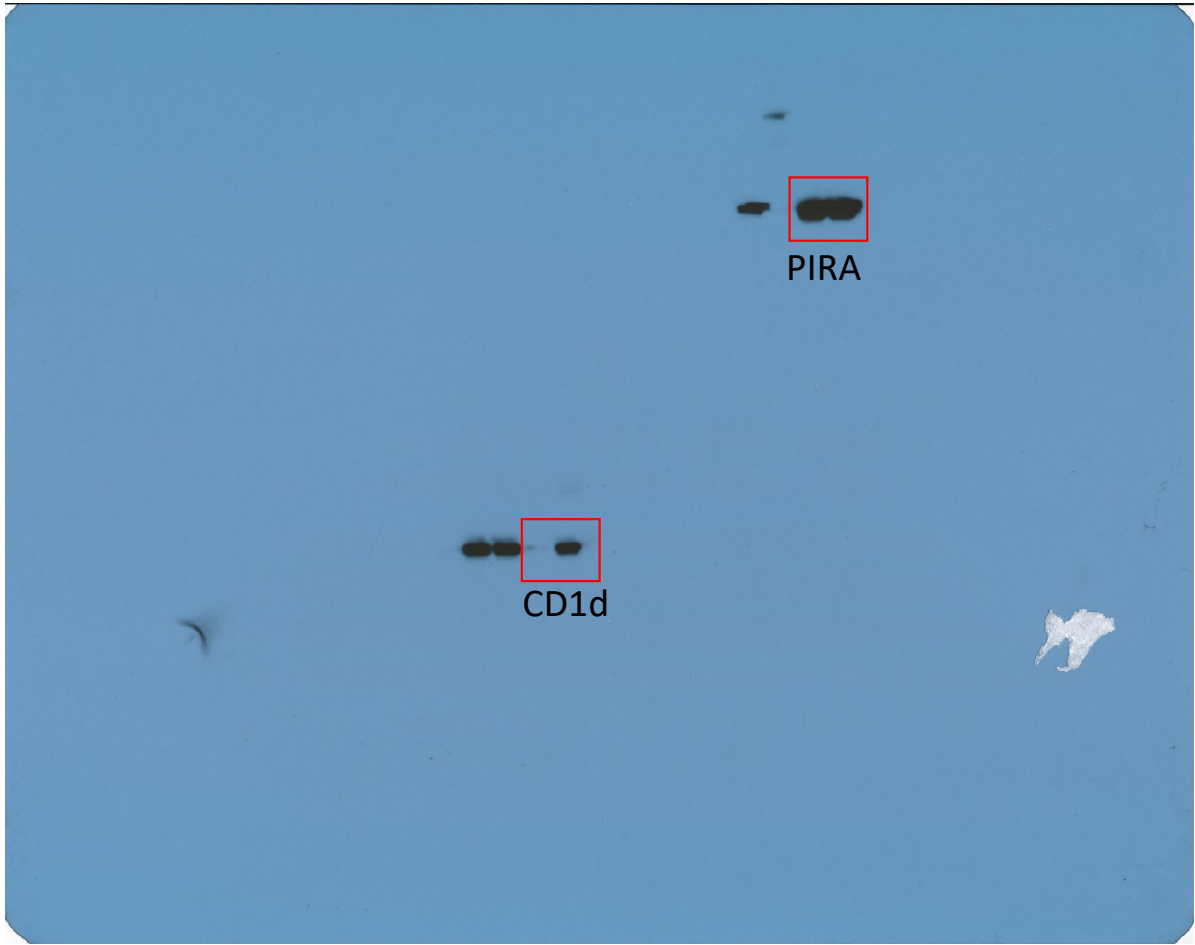

Supplement: Supplementary file 2 — unprocessed wb images [file 41419_2026_8789_MOESM2_ESM.pdf]
